# Impact of a national donor vigilance system: An example of improved donor health by reduction of delayed bleedings after donor plasmapheresis

**DOI:** 10.1111/vox.70307

**Published:** 2026-06-04

**Authors:** Pia Braagaard Hartfelt, Christina Mikkelsen, Bitten Aagaard, Rune Larsen, Sys Hasslund, Louise Ørnskov Drechsler, Mark H. Yazer, Mie Topholm Bruun

**Affiliations:** ^1^ Clinical Immunology Research Unit, Department of Clinical Immunology Odense University Hospital Odense Denmark; ^2^ Department of Clinical Immunology Copenhagen University Hospital Copenhagen Denmark; ^3^ Department of Clinical Immunology Aalborg University Hospital Aalborg Denmark; ^4^ Department of Clinical Immunology Naestved University Hospital Naestved Denmark; ^5^ Department of Clinical Immunology Aarhus University Hospital Aarhus Denmark; ^6^ Department of Pathology University of Pittsburgh Pittsburgh Pennsylvania USA; ^7^ Department of Clinical Research University of Southern Denmark Odense Denmark

**Keywords:** adverse donor reactions, delayed bleeding, donor plasmapheresis, donor safety, donor vigilance

## Abstract

**Background and Objectives:**

In Denmark, all blood banks use the same standardized donor vigilance system, enabling comparisons of adverse reactions across the country's five regions. At several collection sites, a compression bandage is applied over the venipuncture site after donation. In this study, we investigate whether using a compression bandage is more effective than using a non‐compression bandage in preventing delayed bleeding.

**Materials and Methods:**

Data were collected from four of the Danish regions (data from the fifth region were incomplete) between 2021 and 2024 using the Danish donor vigilance system. In one region, compression bandage has been used consistently since 2021, whereas three other regions transitioned from non‐compression bandages to compression bandages at different times during the study period.

**Results:**

Data from 426,250 donor plasmaphereses were analysed; 160,547 (37.7%) were collected without the compression bandage and 265,703 (62.3%) were collected with the bandage. A significantly lower number of delayed bleeding events occurred when the bandage was applied post‐donation (0.3% vs. 0.04%, respectively, *p* < 0.001).

**Conclusion:**

The implementation of the compression bandage resulted in a significant decrease in delayed bleeding in Danish plasma donors. Using a compression bandage may be an easy and cost‐efficient way to improve donation safety.

**Highlights**

- A donor vigilance system is a critical tool for identifying the prevalence of adverse reactions, thereby facilitating their prevention and enhancing the overall conditions for blood donors.
- By using a compression bandage, the risk of delayed bleeding events is reduced significantly in plasma donors.
- Reducing the risk of adverse reactions is important to ensure donor safety and retention.

## INTRODUCTION

Plasma and plasma‐derived medicinal products are essential life‐saving therapies for certain patient populations. In Denmark, there is an ongoing effort to increase the number of donor plasmapheresis procedures to increase plasma collection. The Danish blood bank system, including donor plasmapheresis, is fundamentally based on voluntary, non‐renumerated blood donors. Therefore, donor safety and the prevention of adverse reactions are in particular focus in the blood banks to minimize the risks associated with donations and to ensure appropriate management in the event of an adverse reaction. Fortunately, donor plasmapheresis is generally considered a safe procedure [1, 2]. In Denmark, donors are permitted to donate plasma every 14 days and up to 33 donations per year including other donation types.

In January 2020, a nationwide donor vigilance system, developed by the Danish Hemovigilance Committee, was implemented across all five regions of the country [3]. The Danish national donor vigilance system was founded upon standards established by the International Society of Blood Transfusion (ISBT) and the Association for the Advancement of Blood & Biotherapies (AABB). This system was established to enhance the collection of data on adverse reactions among blood donors, to monitor adverse reactions and to improve donor safety.

One possible adverse reaction following donor plasmapheresis is delayed bleeding. In accordance with Danish haemovigilance practice, delayed bleeding is defined as bleeding from the venipuncture site occurring after the donor has left the chair/bed or the collection site. This definition is not based on a fixed time interval but on timing in relation to when the donor leaves the donation bed. Registration of delayed bleeding events is mandatory if a change of a bandage is required (after leaving the donation bed) or if the donor self‐reports bleeding after leaving the collection site. For the purpose of this study, delayed bleeding therefore referred to any post‐donation bleeding occurring after the donor left the donation bed, as captured through routine haemovigilance reporting.

A Danish study reported that delayed bleeding accounts for approximately 4% of all donor adverse reactions [4].

For several years, the Region of Southern Denmark has used a compression bandage after donor plasmapheresis to prevent delayed bleeding. In contrast, the North Denmark Region, the Central Denmark Region and the Region Zealand, introduced this practice in 2023 and 2024, having previously relied on a simpler non‐compression bandage to arrest any post‐donation bleeding from the venipuncture site. Aside from historical differences in post‐venipuncture bandaging, the protocols for selecting donors and performing plasmapheresis were identical at all blood centres throughout all regions of Denmark.

This study aimed to assess whether the use of a compression bandage was associated with a reduction in delayed bleeding events compared to the traditional non‐compression bandage, using data from the national donor vigilance system.

## MATERIALS AND METHODS

This retrospective cohort study included donor plasmapheresis data from four regions of Denmark, as data from the fifth region were too incomplete to be included in the data analysis. Information regarding donor plasmapheresis and adverse reactions from all Danish blood centres was registered using the blood bank information technology system ‘ProSang’ (Omda, Stockholm, Sweden) by trained staff in the blood banks.

Donor eligibility in Denmark is regulated by national blood donor guidelines. Individuals with known bleeding disorders are permanently deferred from blood and plasma donation. Donors receiving anticoagulant or potent antiplatelet therapy, such as P2Y 12 receptor antagonists, are generally not eligible for plasma donation, as the underlying conditions (e.g., thromboembolic disease or significant cardiovascular disease) typically constitute exclusion criteria or temporary deferral. Consequently, donors in the current cohort are not expected to have been under active anticoagulant or antithrombotic treatment at the time of donation. An exception to this exclusion criterion is donors who take acetylsalicylic acid (aspirin) or non‐steroidal anti‐inflammatory drugs (NSAIDs) as analgesic or for prophylactic treatment, who are permitted to donate plasma by apheresis.

Within the Danish donor vigilance system, all adverse donor reactions, including delayed bleeding, are classified by type, severity level and imputability score according to standardized national haemovigilance criteria. Severity level can be categorized on a scale from 1 to 5, with 5 representing death. Delayed bleeding is typically mild and classified within severity levels 1 and 2. The imputability score is categorized on a scale ranging from ‘unlikely’ to ‘certain’ and is assigned at the time of reporting by trained staff according to predefined criteria. In Danish haemovigilance practice, delayed bleeding from the venipuncture site is generally classified as having ‘certain’ imputability, as it is considered directly related to the donation procedure. However, individual‐level factors such as donor behaviour or other potential confounders are not systematically incorporated into this classification.

We evaluated data from all donor plasmapheresis procedures in the four regions of Denmark over the period 1 January 2021 to 31 December2024. The donors included both women and men aged 17–75 years and both repeat and first‐time donors.

For all donor plasmaphereses in the Region of Southern Denmark during the study period, a cotton ball and the compression bandage ‘latex‐free support and compression bandage 7.5 cm × 4.5 m’ (CPK‐SPORT, Farmaban, Spain) were applied post‐donation, providing compression over the venipuncture site. None of the other three regions used the compression bandage between 2021 and 2022, whereas one region began using it routinely in August 2023 another in November 2023 and the last region started using it routinely in March 2024. In all regions, donors were advised to keep the compression bandage on for 2 h after the donation.

The number of delayed bleeding events in all regions during the period before the compression bandage was routinely employed was compared with the number of delayed bleeding events when compression bandages were used. Additionally, for the three regions that implemented the compression bandage during the study period, the rates of delayed bleeding events were compared between the periods when the compression bandage was not used and when it was routinely used. A *χ* ^2^ test was used to compare the delayed bleeding data with a significance level set at a *p*‐value <0.05 (Microsoft Excel).

## RESULTS

Table 1 presents the demographic characteristics of all of the plasmapheresis donors and those who experienced delayed bleeding after their plasmapheresis procedure.

**TABLE 1:** Characteristics of the plasmapheresis donors.

	All donors	Donors with delayed bleeding	
	Number (%)	Number (%)	*p*‐value
Sex			<0.001
Men	254,933 (59.9)	384 (64.9)	
Women	170,725 (40.1)	208 (35.1)	
Age			
17–18	2686 (0.6)	1 (0.2)	
19–22	19,932 (4.7)	20 (3.4)	
23–29	75,745 (17.8)	90 (15.2)	
30–69	322,467 (75.7)	476 (80.4)	
≥70	5321 (1.2)	15 (2.5)	
Donor			<0.001
First‐time donor	11,872 (2.8)	3 (0.5)	
Repeat donors	413,786 (97.2)	589 (99.5)	

A total of 426,250 donor plasmapheresis procedures were performed during the study period. Compression bandage was used in 265,703 (62.3%) of the donations. There were 592 (0.14%) cases of delayed bleeding reported to the donor vigilance system during this study period. In the group of donors on whom compression bandages were applied, 111 (0.04%) cases of delayed bleeding were reported. Among donors for whom the compression bandage was not applied after donation, delayed bleeding was observed in 481 (0.3%; *p*‐value <0.001) cases. Delayed bleeding events occurred more frequently in male donors (0.15%) compared to female donors (0.12%; *p*‐value <0.001) and was rarely observed in first‐time donors (0.03%) compared to repeat donors (0.14%; *p*‐value <0.001).

A significant reduction in the incidence of delayed bleeding was observed in the three regions that implemented the compression bandage during the study period. Table 2 presents the rates of delayed bleeding with and without compression bandage per 100,000 donations, along with the specific results from the North Denmark Region, the Central Denmark Region and the Region Zealand before and after the procedural change.

**TABLE 2:** Number and rates of delayed bleeding from 2021 to 2024.

	Delayed bleeding	No delayed bleeding	*p*‐value	Rates per 100,000^a^
North Denmark Region			<0.001	
Non‐compression bandage	128	40,366		318.6
Compression bandage	11	13,624		80.7
Central Denmark Region			<0.001	
Non‐compression bandage	239	73,255		325.2
Compression bandage	37	47,758		77.4
Region Zealand			<0.001	
Non‐compression bandage	113	46,405		242.9
Compression bandage	23	28,884		79.6
All donations			<0.001	
Non‐compression bandage	481	160,066		299.0
Compression bandage	111	265,592		41.8

^a^ Calculated before and after the change in bandage type in each of the individual regions.

## DISCUSSION

The results of this study demonstrate that the application of a compression bandage following donor plasmapheresis is associated with a significantly reduced risk of subsequent delayed bleeding events. Reducing the risk of adverse reactions associated with donor plasmapheresis is a critical objective for blood banks, both to ensure donor safety and to encourage voluntary donor participation.

After nearly 5 years of use, the Danish donor vigilance system has accumulated a substantial number of reports. Consequently, the donor vigilance system represents an important and valuable tool for obtaining a comprehensive overview of the prevalence, incidence and distribution of adverse reactions associated with donor plasmapheresis procedures. The system facilitates the identification of regional variations in adverse reaction rates and can be used to uncover discrepancies in procedures and promote the standardization of procedures with the aim of reducing the incidence of adverse reaction rates and enhancing donor health outcomes.

Although delayed bleeding is not a common adverse reaction to donor plasmapheresis, it remains a bothersome complication for donors. For some donors, it may be perceived as overwhelming and suggest that the procedure is potentially unsafe. It is conceivable that adverse reactions may lead some donors to decline subsequent blood donations [5]. Consequently, the application of compression bandages might be an inexpensive and easy way to retain donors.

We observed a higher frequency of delayed bleeding among men and repeat donors. Male donors constituted 59.9% of the study population and their overrepresentation might partly explain why they more frequently had higher rates of delayed bleeding. More importantly, these findings may reflect behavioural factors rather than purely biological differences. Repeat donors without a history of delayed bleeding may be less likely to strictly adhere to post‐donation recommendations, such as avoiding strenuous physical activity and not removing the compression bandage, compared to first‐time donors who may be more cautious. In addition, first‐time donors may under‐report minor adverse reactions due to uncertainty about reporting criteria, which could also contribute to the observed differences.

All procedures included in the study were plasmapheresis donations, involving removal of plasma and thereby a temporary reduction in circulating coagulation factors. This aspect was not specifically accounted for in the study design or analysis, and no individual‐level coagulation data were available. However, it is possible that this small and transient decrease in clotting factors could also have contributed to delayed bleeding. When all donors underwent the same procedure, the potential effect of coagulation factor depletion is assumed to be similar across regions and study periods and therefore unlikely to confound the comparative analyses.

This study has some limitations. A small number of donors were included twice in the analysis because of their transition between age groups during the period from 2021 to 2024. However, each donation was considered a discreet event, as it related to delayed bleeding events, so the effect of one donation would not have had an impact on subsequent donations.

Furthermore, the documentation of adverse reactions is conducted manually rather than through an automated system, rendering it susceptible to human error. Consequently, there remains a possibility that certain adverse reactions were either undocumented or inaccurately recorded. Additionally, the introduction of a new bandage may have heightened attention towards the reporting and registration of delayed bleeding, potentially influencing reporting practices compared to the period preceding this change.

Another limitation of this study is that donor behaviour after donation was not monitored. Thus, it is possible that some donors engaged in strenuous physical activity following donation, thereby potentially increasing their risk of delayed bleeding. However, donors in all regions receive standardized post‐donation advice, including recommendations to avoid heavy physical exertion after donation, although this cause cannot be excluded as a confounding factor in the rate of delayed bleeding.

Furthermore, the dataset did not identify which donors are taking NSAIDs or acetylsalicylic acid, so their effect on delayed bleeding, if any, could not be analysed.

Previous studies have not specifically investigated the most effective type of bandage for preventing delayed bleeding following donor plasmapheresis procedure; therefore, these data on the use of the compression bandage represent an important step forward in preventing this type of adverse reaction.

Our findings revealed that the incidence of delayed bleeding was significantly lower among donors on whom a compression bandage was applied relative to those who were treated with a non‐compression bandage, and this was true even within specific regions of Denmark that implemented this post‐venipuncture bleeding control method during the study period.

The cost of the compression bandage is marginally higher than that of the non‐compression bandage. Nevertheless, the use of compression bandages represents a small, but effective adjustment in the procedure after donor plasmapheresis enhancing donor safety and health outcomes.

## CONFLICT OF INTEREST STATEMENT

M.H.Y. is on the scientific advisory board for Hemanext, and has given paid lectures for Terumo BCT and Grifols. He owns equity in Velico LLC. The other authors do not have any conflicts of interest.

## Data Availability

The datasets generated and analysed during the current study are available from the corresponding author on reasonable request.
